# Photobiomodulation as a treatment for dermatitis caused by chemoradiotherapy for squamous cell anal carcinoma: case report and literature review

**DOI:** 10.1186/s13014-022-02015-4

**Published:** 2022-03-07

**Authors:** Fabiana Hottz, Daniel Herchenhorn, Juliana Lenzi, Juliana Andrade, Vinicius Freire, Pedro Pinho

**Affiliations:** 1 Department of Physiotherapy, Oncologia D’Or, Rio de Janeiro, Brazil; 2 Instituto D’Or de Pesquisa e Ensino (IDOR), Rio de Janeiro, Brazil; 3 Department of Physiotherapy, Pelvien Clinic, Campinas, Brazil; 4 Department of Oncology, Oncologia D’Or, Rio de Janeiro, Brazil; 5 Department of Radiation Oncology, Oncologia D’Or, Rio de Janeiro, Brazil

**Keywords:** Anal cancer, Radiotherapy, Laser therapy, Radiodermatitis

## Abstract

In-field dermatitis is a severe and common adverse effect of radiation therapy, that can cause significant pain and treatment interruptions in patients with squamous cell anal carcinoma (SCAC) being treated with radical chemoradiation protocols. There are no established therapies for the treatment of radiation induced dermatitis. Photobiomodulation (PBM) is an effective and low-cost treatment for radiation induced mucositis, but have recently been explored to treat in-field dermatitis. We present a case report of the successful use of PBM for the treatment of dermatitis in the anal area in a patient with SCAC treated with concomitant chemoradiation with curative intent and follow with a literature review of the recent advances and possibilities of the use of PBM as a promising strategy. PBM therapy proved to be efficient in the radiodermatitis treatment, both in relieving the symptoms and controlling dermatitis, in addition to improving the patient's quality of life.

## Introduction

Squamous cell anal carcinoma (SCAC) is considered a rare tumor type. The Human papillomavirus infection (HPV infection) is the major risk factor, especially the HPV subtype 16. Currently, the first-line treatment with curative intent consists of chemotherapy (CT) and radiation therapy (RT) combined, followed by perineal surgery only as a salvage therapy in case of relapse or residual tumors [1–3].

The intensity-modulated radiation therapy (IMRT) is the chosen radiation therapy type for treating cancer of the anal canal due to its advanced technology that reduces exposure of healthy tissues to radiation, preserving the patient’s bladder, intestine and sexual function [4–6].

Despite technological advances there are still significant side effects that can cause morbidity and interfere with treatment, causing pain and also treatment interruptions, such as in-field dermatitis. Singh and cols. demonstrated the frequency of dermatitis in patients undergoing RT in the breast which may affect as much as 95% of the patients. The intensity varies from mild erythema to dry or wet desquamation, which can affect the quality of life and cause treatment delays [8].

Radiation induced dermatitis derives to the fact that the target is closer to the skin and receives a high radiation dose [9].

Total radiation dose, dose fractionation scheme, type of external beam used, radiosensitivity, concomitant chemotherapy, volume and area to be treated can be associated risk factors [7].

Acute radiodermatitis in anal canal cancer is considered to be highly prevalent, occurs in 99.1%. Severe acute radiodermatitis cancer of the anal canal has an incidence rate of approximately 34.5% and may lead to treatment interruption [10, 11].

Treatment of radiodermatitis is not well established. Skin care should be advised to prevent injury and infection from reaching a greater grade [12, 13].

PBM, is a treatment already mentioned in studies for preventing and controlling radiodermatitis in patients undergoing RT for breast cancer is PBM, which is appeared safe, non-invasive, low-cost resource, having already been widely used in head and neck tumors to prevent mucositis, morbidity that impairs functionality and negatively impacts quality of life, in addition to increasing treatment costs [14–16].

Prevention of OM in patients undergoing RT for head and neck tumors suggest that PBM does not interfere with the tumor or the results of treatment and overall survival [17, 18].

This study presents a case of PBM application related to radiotherapy in a patient undergoing combined chemoradiotherapy for squamous cell anal canal carcinoma and it also reviews previous studies using this therapy as treatment and/or prevention of skin reactions caused by radiotherapy.

## Case report

A 62-year-old man, presented a lesion in the anal and perianal region, followed by weight loss, tenesmus, urgency and discrete fecal incontinence, and was diagnosed with cancer of anus and anal canal, identified as invasive squamous cell carcinoma (SCAC) in October 2019. After a magnetic resonance imaging (MRI) of the abdomen and pelvis, in addition to computed tomography of the chest, he was staged II.

The patient underwent treatment with capecitabine at a dose of 850 mg/m^2^ bid during days of radiotherapy (tumor bed and drainage chains). The choice of capecitabine was due to his recent HIV diagnosis and the concomitant use of antiretroviral therapies. IMRT was performed in a total of 30 fractions, energy (photon beam), RapidArc™ technique, 6MV, 5400 cGy, totaling 05 weeks of treatment (10/22/2019 to 12/12/2019) to the anal lesion and pelvic nodes.

**Fig. 1:**
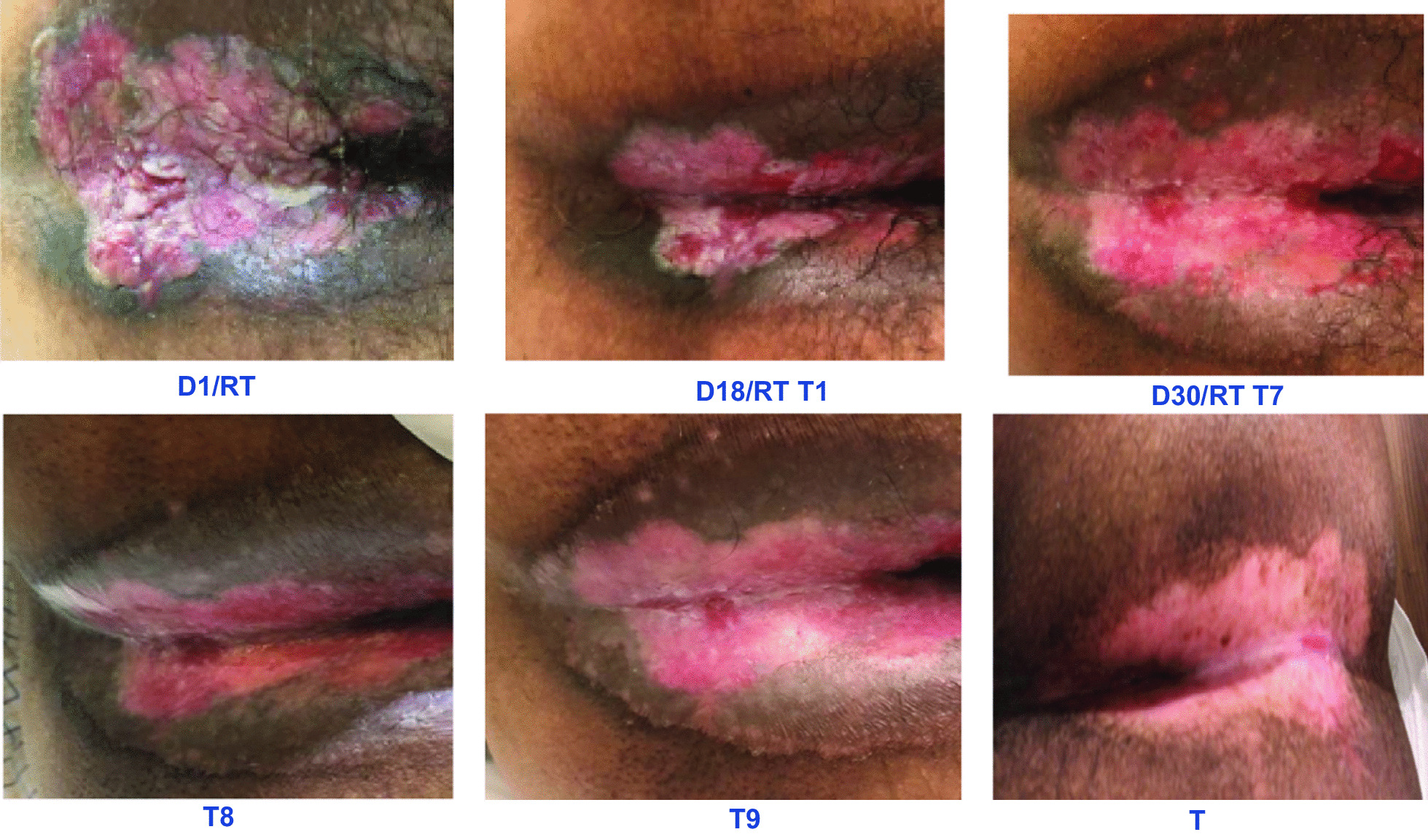
Radiodermatitis (RD) in an 62-year-old man, diagnosed with cancer of anus and anal canal, identified as invasive squamous cell carcinoma (SCAC), stage II, in October. The patient referred weight loss, tenesmus, urgency and discrete fecal incontinence. D1-RT: First visit to the physiotherapy; tumor wound, wasn’t performe the PBM. VAS = 0. D18-RT, T1: First PBM. VAS = 9; tumor wound control; burning in the anal area; RTOG (grade III). D30-RT, T7: seventh PBM. Last day of the RT. VAS = 3; burning slight in the anal area; RTOG (grade III). T8: eighth PMB session five days after the end of radiotherapy. VAS = 0; asymptomatic; RTOG (grade II/III). T9: ninth PBM session seven days after the end of radiotherapy. VAS = 0; asymptomatic; RTOG (grade II). T: Follow-up one year after the end of PBM sessions. VAS = 0; current; RTOG (grade 0)

He was referred to physiotherapy before starting combined therapy. Upon examination, it was found the presence of an anal fistula and perianal tumor wound (Fig. 1) with no associated pain. On D18 of RT he presented with a grade 3 perianal radiodermatitis, according to the Radiation Therapy Oncology Group (RTOG) criteria, which was related to radiotherapy. He complained of burning pain in the anal mucosa when defecating, and there was an increase in defecatory frequency with a predominance of pasty to liquid stools, with a 6/7 classification on the Bristol scale [9, 19].

PBM application with low-level laser (DMC EC POTENCIA brand; 100 MW, spot size = 0.03 mm) was used, in red light length, with a dose of 2 J (joules) of energy in the irradiated perianal area and anal region twice a week—with an interval of 48 h between sessions for 4 weeks, until D7 after RT completion, which resulted in a relieve of pain and burning symptoms when defecating, evaluated by the visual analog scale (VAS) of pain from 9 to 3, and decrease of the radiodermatitis grade from 3 to 2. The relief of the symptoms of radiodermatitis happened in the first application, in the seventh application (T7) of PBM, the patient referred VAS 3, and from eighty application (T8), asymptomatic.

Of note, patients using antiretroviral therapy seem to have greater toxicity to the treatment and may present greater toxicity on their skin and gastrointestinal when compared to patients not infected with HIV. Occasionally, they need to suspend or decrease radiotherapy dose [20, 21].

## Discussion

Anal canal cancer is relatively rare. Radiation therapy is often the chosen modality for treating this disease. Radiodermatitis is one of the side effects of radiation therapy that can interfere with treatment adherence, and also a cause of serve pain and morbidity during and after therapy. It is a tissue inflammatory response that can progress to ulceration or tissue necrosis [22, 23].

In fact, Aragüés and colleagues suggest that acute radiodermatitis appears between 10 and 14 days after radiation therapy. However, radiodermatitis cases have been decreasing due to better treatment planning and the use IMRT. According to Han and collaborators, RD is considered the most common acute adverse effect of RT, with a frequency between 10%, 46% and 57% in anal and perianal cancer. In another retrospective study on breast cancer, the incidence of radiodermatitis was 81.19% and score 2 more prevalent [24, 25].

A prospective study, carried out by Kachnic et al. [26] evaluated the dose-painted intensity-modulated radiation therapy (DP-IMRT) method, in which it allows the allocation of different dose targets for the treatment, creating a different dose distribution for each location, as a result, Radiation Therapy Oncology Group (RTOG) 0529, showed shorter treatment interruption and significant savings in grade 3 acute dermatology toxicities (49%) compared to RTOG 9811 (23%), without using DP-IMRT, in addition to showing that 45% of patients discontinued treatment for acute dermatitis, of these, 88% duo to pain.

Chronic radiodermatitis can appear approximately 90 days after radiotherapy and tissue repair chronification process induces fibrosis formation, and consequently, it can be followed by as pain, evacuating difficulty, pelvic dyssynergy and anal canal stenosis [2, 8].

Radiodermatitis development mechanism is largely linked to the inflammatory response associated with oxidative stress. Cellular damage induced by radiation, especially in the mitotic phase, triggers an inflammatory cascade that, when it becomes chronic associated with oxidative stress, leads to a modification of cytokines, alteration of the cell cycle and also promotes DNA damage. These changes support the inflammatory cascade, and consequently lead to disorderly tissue repair [27].

Currently, radiodermatitis treatment and prevention is based on polytherapies individualized to each service. Literature has shown effectiveness to mitigate radiodermatitis grade in breast cancer [28]. Robijins et al. [29] demonstrated that PBM can prevent acute radiodermatitis in patients with breast cancer submitted to RT. Additionally, that study, demonstrated that 12% of the patients in the treatment group had radiodermatitis grade 2, while 44.4% of the patients in the control group had radiodermatitis grade 2 or greater grades, concluding that the severity of skin reactions were significantly less in the group which PBM was performed, which means that this is an effective tool for preventing acute radiodermatitis, thus improving the quality of life of the patients.

In other study, the Dermishead trial, Robijins et al., Selected 46 head and neck cancer patients who underwent radiotherapy (RT) with or without concomitant chemotherapy to receive PBM or placebo treatment and investigate if PBM could be effective. As a result, 77.8% of the patients in the control group had grade 2–3 radiodermatitis compared to 28.3% of the PBM. There was a 49% reduction in severe radiodermatitis in the PBM group. This randomized study demonstrated the effectiveness of PBM for the prevention and management of radiodermatitis.

There are only two reported cases of low-level laser application to treat radiodermatitis in the anal area. The first one described in the laser approach for chronic radiodermatitis and the second one was described for the treatment of radiodermatitis in rectal cancer during RT. In both cases there was a noticeable relieve in pain symptoms in the perianal region and tissue mucosa, allowing the patient to return to their daily activities [30].

A randomized prospective study evaluated patients with head and neck cancer and showed that the use of PBM to treat oral mucositis (OM) was associated to a higher complete response rate to treatment. Patients who were followed for 40.3 months had a statistically greater complete response in the PBM group compared to the placebo group (89.1% vs. 67.4%), in addition to an increase in progression-free survival (61.7% vs. 40.4%) and a tendency for better overall survival (57.4% vs. 40.4%). Patients who received PBM had a lower incidence of grade 3–4 OM (6.3% vs. 48%), thus, reduced gastrostomy, less interruption of treatment and use of opioids. In addition to having a positive impact on therapy adverse-events and a major impact on quality of life, positive results in response and survival reinforce the use of this therapy as part of the multidisciplinary approach for patients with head and neck cancer [31].

Radiodermatitis, in addition to severely affecting the patient’s quality of life, can also cause a radiotherapy treatment interruption. Therefore, an effective approach is needed to treat and prevent this common effect.

PBM promotes tissue regeneration due its cellular and anti-inflammatory biomodulation action, relieving pain and promoting healing [32]. This case report reinforces the results of the aforementioned studies. The use of low-level laser with red light length is effective in the control of radiodermatitis.

Despite the limitations above, our approach was satisfactory, since there was a pain relief on the visual analog scale, tissue healing and reduction of radiodermatitis according to the classification of RTOG from grade 3 to grade 2 in the anal region in four weeks. PBM is an effective, safe and low-cost therapeutic resource and not interfere with therapy efficacy.

## Conclusion

PBM in the anal region during RT treatment enabled symptom relief, radiodermatitis control and improved the patient's quality of life. In addition to being an innovative, safe and low-cost therapeutic option. We believe there is a need randomized clinical trials to better define the parameters and introduce this resource as a treatment protocol for radiodermatitis in the anal region.

## Data Availability

Department of Radiation Therapy, Oncologia D’Or, Rio de Janeiro, Brazil.

## Abbreviations

CT: Chemotherapy
DP-IMRT: Dose-painted intensity-modulated radiation therapy
HPV infection: Human papillomavirus infection
IMRT: Intensity-modulated radiation therapy
MRI: Magnetic resonance imaging
OM: Oral mucositis
PBM: Photobiomodulation
RT: Radiation therapy
RTOG: Radiation Therapy Oncology Group
SCAC: Squamous cell anal carcinoma
VAS: Visual analog scale
